# The Reproductive Toxicity Associated with *Dodonaea viscosa*, a Folk Medicinal Plant in Saudi Arabia

**DOI:** 10.1155/2021/6689110

**Published:** 2021-01-15

**Authors:** Muhammad Farooq Khan, Ali S. Alqahtani, Omer M. Almarfadi, Riaz Ullah, Fahd A. Nasr, Omar M. Noman, Nasir A. Siddiqui, Abdelaaty A. Shahat, Syed Rizwan Ahamad

**Affiliations:** ^1^Department of Zoology, College of Science, King Saud University, P.O Box 2455, Riyadh 11451, Saudi Arabia; ^2^Department of Pharmacognosy (Medicinal, Aromatic and Poisonous Plants Research Center), College of Pharmacy, King Saud University, P.O. Box 2457, Riyadh 11451, Saudi Arabia; ^3^Central Laboratory, College of Pharmacy, King Saud University, Riyadh 11451, Saudi Arabia

## Abstract

*Dodonaea viscosa* is a medicinal plant which is being used to treat various diseases in humans. The available safety data suggest that the plant does not produce any side effects, or toxicity, in tested adult experimental animals. However, the influence of *D. viscosa* on fetus or embryonic development is largely not known. This study was conducted in order to find out the reproductive toxicity of *D. viscosa* in experimental animals. Zebrafish embryos were used as the *in vivo* developmental toxicity animal model. Methanolic crude extract, hexane, chloroform, and butanol fractions were prepared from the leaves of *D. viscosa*. Zebrafish embryos were exposed to serial dilution of crude extract and other fractions. The crude extract and hexane fraction induced higher level of toxicity in zebrafish embryos as compared to chloroform and butanol fractions. The phenol and flavonoid estimation revealed that crude leaves extract and hexane fractions had lower content of phenol and flavonoid. Two major compounds, phytol and methyl ester, of hexadecanoic acid were identified by gas chromatography and mass spectrophotometry (GC-MS) analysis. More detailed studies are needed to check the toxicity of *D. viscosa* in pregnant experimental animals; however, the results from this study have shown that *D. viscosa* possesses reproductive toxicity and its use and doses must be carefully monitored in pregnant patients.

## 1. Introduction

In recent years, there has been a renewed interest into the biological activity of traditional plant medicines, and the role of natural products in drug discovery [1–3]. Reasons for this include the great need for new molecular models, as this leads to potential new drugs, and for authenticating traditional applications for use in current therapy [4]. *Dodonaea viscosa* is an evergreen shrub belonging to the Sapindaceae family that consists of about 150 genera and 200 species [5]. Initially, it was a native of Australia and later widespread throughout the tropical regions [6]. In Saudi Arabia, *D. viscosa* is widely found in the southern province, Hijaz region, and eastern region [7]. It has been accounted as one of the famous traditional remedies for wide verities of ailments like rheumatism, fractures, diarrheas, gout, hepatic or splenic pain, smooth muscles disorders (uterine pain), hemorrhoids, snake bites, and sore throat [8]. The literature review has shown that ethanol extract of *D. viscosa* flower exhibits cytotoxic activity against breast cancer in an *in vitro* study [9]. Moreover, the anticancer activity of the ethanol extract through induction of apoptosis in human breast cancer cell line has been reported [10]. In addition, dodonaeasides A and B, triterpenoid saponins, were isolated from ethyl acetate extract of *D. viscosa* roots and showed antiproliferative property against human ovarian cancer cell line [11]. Besides, the literature review revealed other biological activities of *D. viscosa* such as antimicrobial, antioxidant [12], anti-inflammatory [13], analgesic, antipyretic [14], antifungal [15], gastroprotective [16], antimalarial [17], and antispasmodic activities [18]. Furthermore, phytochemical screening has shown that *D. viscosa* contains flavonoids, alkaloids, terpenoids, saponins, sugars, and tannins [19]. Due to the diverse biological properties *D. viscosa* possesses, various remedies prepared from it are routinely used by humans to cure many ailments. Some studies have reported that *D. viscosa* has not produced severe toxicity in experimental animals; however, the toxicity of *D. viscosa* on developing fetus in human, or in other experimental animals, is largely not known. Therefore, this study was designed to investigate the developmental toxicity of various extracts prepared from the leaves of *D. viscosa* in zebrafish embryos.

## 2. Materials and Methods

### 2.1. Plant Collection

Fresh leaves of *D. viscosa* (Sapindaceae) were collected from the Herbal Garden, College of Pharmacy, King Saud University, Riyadh, Saudi Arabia, and botanically identified by Dr. Mohammed Yusuf, Field Taxonomist, Department of Pharmacognosy, College of Pharmacy, King Saud University. A voucher specimen (voucher #15787) was deposited at the herbarium of Pharmacognosy Department, College of pharmacy, KSU, Riyadh, KSA.

### 2.2. Extraction

428 grams of air-dried leaves was ground to a coarse powder using an electrical grinding mill and extracted by cold maceration in 1200 mL 80% methanol (Sigma-Aldrich, MO, USA), at room temperature and allowed to stand overnight for 3-4 days with occasional shaking to produce crud extract; this process was repeated 3 successive times. Then, the crude extract was filtered using Whatman filter paper No. 1 (Wagtech International Ltd., England) and concentrated using a rotary vacuum evaporator (Buchi Rotavapor) under reduced pressure at 45 rpm and controlled temperature (40°C). After drying, a total of dry crud extracts, 133 grams, was kept in the refrigerator until use.

### 2.3. Solvent Fractionation

128 grams of the crude extract was subjected to fractionation fractions using increasing polarity organic solvents purchased from Sigma-Aldrich, MO, USA (i.e., *n*-hexane, chloroform, ethyl acetate, and *n*-butanol) successively. It was transferred to a separatory funnel and suspended in 475 mL of distilled water and partitioned with 3 × 500 mL of each solvent. The filtrate was concentrated using a rotary vacuum evaporator under reduced pressure at 45 rpm and 40°C. The yield of the dried fractions was 0.86%, 27.56%, and 23.95% for the *n*-hexane, chloroform, and *n*-butanol fractions, respectively. Then, the dried fractions were transferred into separate vials and stored at −20°C until use.

## 3. Phytochemical Analysis

### 3.1. GC-MS Analysis and Compound Identification

The chemical constituents of *D. viscosa* hexane fraction (DVHF) were determined using gas chromatography and a mass spectrometer (Turbomass, PerkinElmer, Inc., Waltham, MA, USA). The temperature of program 4 was set to 40°C, followed by a 2 min hold, and then raised to 200°C at a rate of 5°C min^−1^, which was also then put on hold for 2 min. From 200°C, the temperature was raised by 5°C min^−1^ to 300°C and held for another 2 min. The mass spectra of DVHF compounds were also compared with those of similar compounds in the Adams Library [20] and the Wiley GC/MS Library [21].

### 3.2. Estimation of Total Phenol and Flavonoid Contents

Total phenolic content of *D. viscosa* extract and fractions was determined by the standard Folin–Ciocâlteu spectrophotometric method [22, 23]. Briefly, 0.5 mL of each extract was added to 0.1 mL of Folin–Ciocâlteu reagent (0.5 N) and the contents of the flask were mixed thoroughly. Later, 2.5 mL of sodium carbonate (Na_2_CO_3_) was added, mixed, and incubated for 0.5 h. The optical density was measured at 760 nm utilizing UV-visible spectrophotometer. The total phenolic contents were expressed as mg gallic acid equivalents (GAE)/g of the extract. For total flavonoids content, standard aluminium chloride spectrophotometric method according to [24] was used. In brief, 1 mL of each extract at a concentration of 1 mg/mL was taken. Then, 1 mL of AlCl_3_ (10%) was added sequentially. The test solution was vigorously shaken. Absorbance was recorded at 415 nm after 30 minutes of incubation.

### 3.3. Animal Test

Zebrafish embryos less than 5 days after fertilization were used in this study and hence exempted from taking approval from institutional ethical committee for the care and use of laboratory animals as stated in [25]. Maintenance and breeding procedures were performed by following the guidelines mentioned in zebrafish book [26]. Embryos were obtained after pairwise mating of adult fish in one-liter sterile breeding tanks by putting two females and one male. Embryos were collected and washed using embryo medium fertilized and dead embryos were separated according to the descriptions of Kimmel et al. [27].

### 3.4. Embryo Short-Term Toxicity Test

Fertilized embryos were transferred into 6-well plates (30 embryos/well) containing a series of diluted extracts ranging from 0.15 *µ*g/mL to 300 *µ*g/mL in total volume of 2 mL and incubated overnight in an air incubator at 29°C. Embryo development was monitored first after overnight and then after every 24 h interval for 96 h. Four morphological characteristics were evaluated including coagulation of eggs, tail detachment, presence of heart beat, and hatching rates using Carl-Zeiss Inverted Microscope (observer D1) equipped with differential interference contrast (DIC) filter and camera and software for image optimization. Experiments were conducted in triplicate.

### 3.5. Calculation of LC50

Median inhibitory concentration (IC_50_) and median lethal concentration (LC_50_) were statistically analyzed by using the Probit analysis using Excel spread sheet as stated in [28].

### 3.6. Acridine Orange Staining in Whole Mount

Apoptosis was analyzed in hexane extract of *D. viscosa* treated whole-mount embryos with an exposure to the sub-lethal concentration of 0.5 *μ*g/mL at 24 hpf (hours post fertilization). Control and treated embryos were rinsed several times with distilled water and then exposed to a solution of acridine orange (Carlo Erba) at a concentration of 100 *μ*g/mL for 1 h, after which several rinses were performed to remove the excess dye. The embryos were then mounted in a drop slide with glycerol and PBS. The images were acquired with Olympus SZ10 florescent microscope using FITC channel.

## 4. Results and Discussion

### 4.1. DVHF Chemical Composition

**Figure 1:**
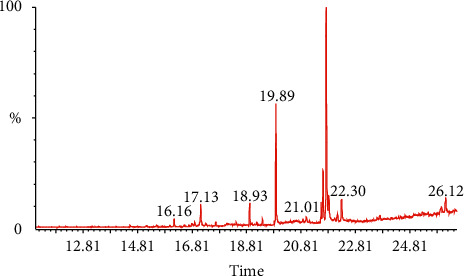
GC-MS analysis of DVHF.

**Figure 2:**
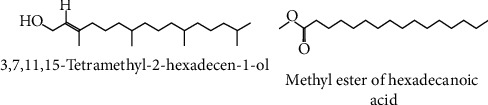
Major active constituents of DVHF.

Figure 1 shows the retention times and area percentages of DVHF. In Table 1, the identified compounds are represented in order of their elution on the HP Innowax column. A total of 9 compounds, representing 98.99% of the total extract, could be identified. 3,7,11,15-Tetramethyl-2-hexadecen-1-ol (52.7%) (Figure 2) was the primary constituent, and methyl ester of hexadecanoic acid (26.3%) (Figure 2) was a moderate constituent. The other compounds are present in fairly good amounts.

### 4.2. Estimation of Total Phenol and Flavonoid Contents

**Figure 3:**
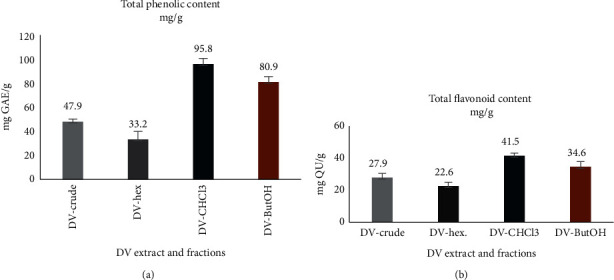
(a) Total phenolic content and (b) total flavonoid content of *D. viscosa* extract and fractions.

The total phenolic content of the *D. viscosa* extract and fractions was measured with the Folin–Ciocâlteu reagent assay and the results are shown in Figure 3(a). The values varied from 33.2 to 95.8 mg gallic acid/g of dry extract. Chloroform fraction contained the highest amount of phenolics (95.8 mg gallic acid/g), followed by butanol fraction (80.9 mg gallic acid/g), whereas the lowest level was found in the hexane fraction (33.2 mg gallic acid/g). Total flavonoid content of *D. viscosa* extract and fractions was measured with standard aluminium chloride spectrophotometric method and the results are shown in Figure 3(b). The values varied from 22.6 to 41.5 mg quercetin/g of dry extract. It was shown that chloroform fraction contained the highest total flavonoid content (41.5 mg QU/g), followed by butanol fraction (33.6 mg QU/g), whereas the lowest level was found in the hexane fraction (22.6 mg QU/g).

### 4.3. *D. Viscosa* Induced Severe Toxicity and Teratogenicity in Zebrafish Embryos

**Figure 4:**
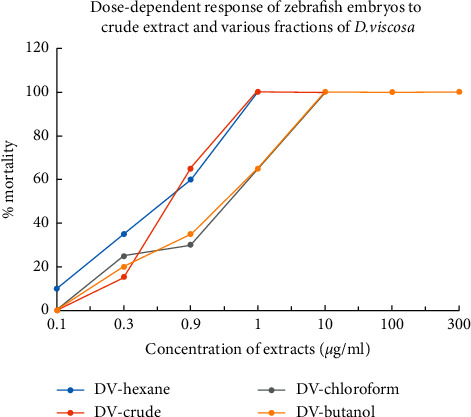
Dose-dependent response of zebrafish embryos on mortality exposed to crude extract and various fractions of *D. viscosa*.

**Figure 5:**
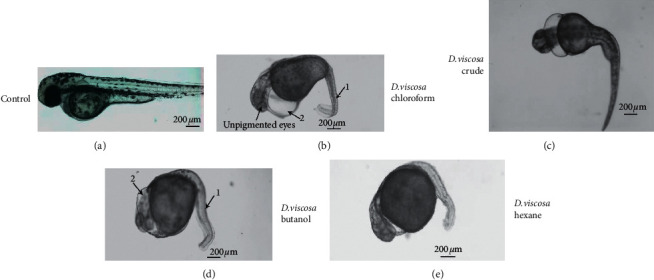
*D. viscosa* induced severe teratogenicity in zebrafish embryos. Representative micrograph of live zebrafish embryos mock treated (control) and treated with crude extract and various fractions of *D. viscosa*. The images were recorded after two days of exposure to zebrafish embryos to sub-lethal concentration of crude and various fractions. The images are arranged in order from mild to severe abnormalities, being the crude extract. The presence of pectoral fin mock treated embryos shows that the embryos are not developmentally delayed. The mock treated embryos have straight body with straight arrangement of notochord (black arrow, notochord in control) and pigments all around the body and also have pigment development in eyes, whereas the treated embryos lack the pigmentation and their body is curved due to abnormal development of notochord (represented by “1” in treated embryos). The treated embryos have large cardiac edema (2 in all the treated embryos). The pigment is also lacking in treated embryos except the embryos which were treated with crude extract.

**Figure 6:**
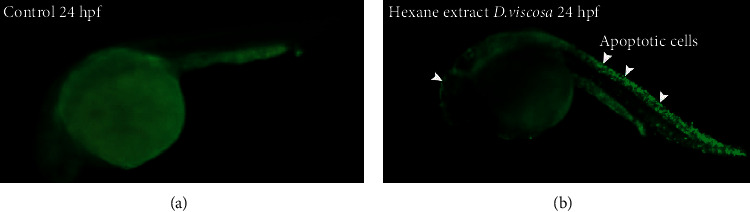
The hexane fraction of leaves extract of *D. viscosa* induced severe level of apoptosis in zebrafish embryos at 24 hpf. The level of apoptosis was evaluated in live zebrafish embryos which were treated with hexane fraction of *D. viscosa*. The hexane fraction induced severe embryonic toxicity; hence, the embryos treated with hexane fraction are shown. The live embryos are exposed to acridine orange as described in the methodology section. The images are taken by fluorescence microscope under FITC filter. Not many acridine orange positive (green) cells are observed in control (left image), whereas a lot of acridine positive cells are observed in zebrafish embryos which were treated with hexane fraction.

The *in vivo* toxicity screening performed on zebrafish revealed that *D. viscosa* extracts were toxic towards the embryo's development and survival (Figure 4). The survival rate and effect on embryonic development were carried out first after overnight exposure and then every 24 h by optical monitoring. The values obtained thus have proved the dose-dependent effects of *D. viscosa* extracts on embryos mortality and survival curves during the 96 h. As shown in Figure 4 and Table 2, severe toxicity and teratogenic effects were observed in zebrafish embryos which were treated with either crude extracts or various fraction of *D. viscosa.* The *D. viscosa* extracts turned out to be very toxic to zebrafish embryos at concentration less than micrograms and kill the treated embryos within 12 hours of exposure. The hexane fraction was most toxic with LD_50_ values of 0.589 ± 0.30 µg/mL followed by crude extract with LD_50_ values 0.684 ± 0.74. The LD_50_ values for butanol and chloroform fractions were 3.139 ± 0.57 and 3.953 ± 0.43 µg/mL. The zebrafish embryos which were treated with sub-lethal concentration (less than LD_50_ values) exhibited severe embryonic abnormalities.  Figure 5 shows the representative micrograph of control and treated embryos showing the teratogenic effect. As shown in Figure 5(a), the morphological features of mock treated embryos revealed a straight notochord, round yolk sac and pigmentation on the body and eyes, and normal heart shape and rate. The presence of pectoral fin in mock treated embryos at 72 h [27] verifies that the embryonic development in mock treated embryos is not hampered. The mock treated embryos also hatched normally ≤48 hpf. The zebrafish embryos treated with sub-lethal concentration (0.5 *µ*g/mL) of crude extract of *D. viscosa* had bent spine, a large cardiac edema, and severe developmental delay as compared with mock treated counterpart (Figure 5(b)). The treated embryos did not hatch ≤48 hpf. The zebrafish embryos treated with sub-lethal concentration (3.00 *µ*g/mL) of chloroform extract were mildly affected; however, the treated embryos had bent spine, large cardiac edema and cardiac looping (both chambers of the heart get connected with each other), and slow heart rate as compared to control embryos. The developmental stage criteria revealed a mild developmental delay in chloroform extract treated embryos; the presence of pectoral fin bud in these embryos showed that they are at 48 hpf of developmental stage. The zebrafish embryos treated with hexane (Figure 5(e)) or butanol (Figure 5(d)) extract showed severe developmental abnormalities. The treated embryos had bent spine, absence of pigmentation, large yolk sac, and cardiac edema. The embryos were severely developmentally delayed as compared to their mock treated counterpart. The undeveloped heart was observed in both of the extracts treated embryos. These embryos never hatched and also severe apoptosis was observed in these embryos as revealed by acridine orange staining (Figure 6).

## 5. Discussion

Any drug or herbal formula which is intended to be used in humans must first be tested in suitable experimental animals in order to evaluate its safety. *D. viscosa* is being used in many traditional herbal formulas. Even though there are not many toxicological studies, the available data suggests that *D. viscosa* did not induce toxicity in experimental animals. The ethanolic extract prepared from the leaves of *D. viscosa* inhibited the carrageenin induced paw inflammation and also did not cause any toxicity in mice [13]. In another study, no toxicological signs were noticed at a dose of 1250 mg/kg in rats [16]. In a dermotoxicity test, no irritation was noticed by 80% methanol extract of the leaves of *D. viscosa* in skin of mice and rats [29].

The embryos and infants are highly sensitive to chemicals that could cause serious damage to growth during development. The reproductive toxicity of various extracts of *D. viscosa* on embryonic development has never been investigated before this study. We used zebrafish embryos to find out the developmental toxicity of *D. viscosa.* Zebrafish embryos are being used successfully in many developmental toxicity testing screens. Using zebrafish in developmental toxicology has many advantages over other experimental animal model systems. Zebrafish embryos grow and hatch rapidly and have high fertility, and the small and transparent body is suitable for observation of internal organs with conventional microscopy. Large-scale screening in 96-well plate is possible for high-throughput chemical screens with zebrafish embryos. Moreover, easy exposure of embryos to chemicals with minimal amounts makes it an ideal model organism to test the developmental toxicity of chemical or extracts on a large scale [30–33]. The ex utero embryonic development taking place allows the observation of embryonic development without surgical intervention [34].

The most prominent toxic effect of *D. viscosa* on zebrafish embryos (with almost all the extracts) was egg coagulation or mortality within few hours of exposure. 100% of treated embryos died within 6 hours of exposure at concentration in microgram range. The most toxic extract was hexane fraction which induced 100% mortality in treated embryos ≤0.7 *µ*g/mL followed by the crude extract where 100% mortality was observed with concentration ≤1.00 *µ*g/mL.

The phenol and flavonoid content of different extracts revealed that the hexane fraction of *D. viscosa* had the lowest phenol and flavonoid contents. The hexane fraction had phenol and flavonoid content of 33.2 and 22.6 mg/mL, respectively, whereas the crude extract showed 47.9 and 27.9 mg/mL of phenol and flavonoid contents. The chloroform and butanol extract also exerted toxicity in treated zebrafish embryos, albeit at a higher concentration as compared to hexane and crude extract. The toxicity of the extracts of *D. viscosa* in zebrafish embryos has shown a direct relationship with the amount of phenol and flavonoid. The crude extract and hexane fraction induced the highest level of toxicity and contained the least amount of phenol and flavonoid as compared to other fractions. A similar kind of observation has been reported previously with other medicinal plants. The developmental toxicity of four different types of herbal plants (*Andrographis paniculata, Cinnamon zeylanicum, Curcuma xanthorrhiza, Eugenia polyantha* and *Orthosiphon stamineus)* was investigated in zebrafish embryos. It was shown that the extracts with low contents of phenol and flavonoid induced higher level of toxicity in zebrafish embryos [35].

It is well documented that there is direct relationship between phenolic and flavonoid contents and antioxidant activity of herbal extracts [36, 37]. In one study, the antioxidant (free radical scavenging) activity in *D. viscosa* has been shown to be directly dependent on content of phenol flavonoid. The methanol extract of *D. viscosa* showed 50.72% free radical scavenging activity at 50 *μ*g/mL of phenol and it increased to 92.45% at 1000 *μ*g/mL [12]. Similarly, another study reported 82% of radical scavenging inhibition activity with 300 *μ*l while 80% with 100 *µ*l of flower extract of *D. viscosa* [38].

The imbalance between the production of reactive oxygen species (ROS) and the antioxidant defenses caused pathogenesis of a variety of diseases. It is reported that, in newborns, inadequate antioxidant enzymes such as glutathione stores, and nutritional antioxidants elevate oxidative stress which would affect the development and growth of many organs [39]. It is quite likely that the embryonic abnormalities which have been observed in zebrafish embryos upon treatment of hexane fraction of *D. viscosa* leaves could be due to the elevated level of oxidative stress in these embryos.

The crude extract and various fractions of *D. viscosa* induced severe embryonic teratogenic effect in zebrafish embryos when treated with lower than LD_50_ concentration. Zebrafish embryos which were treated with crude extract or fractions exhibited severe developmental delay and did not hatch. The delayed hatching is supposed to be due to the absence of activity of zebrafish hatching enzyme 1 (ZHE1) which is secreted by hatching gland of zebrafish [40].

The methanolic extract of the leaves of *D. viscosa* has been shown to possess the antifertility activity in female rats. Reduction in the number of liters and early abortifacient and anti-implantation activity had also been observed in female rats which were treated with methanolic leaves extract of *D. viscosa* [41]. The infertility activity of *D. viscosa* extracts has not been investigated in adult zebrafish; however, the teratogenicity and delayed hatching of zebrafish embryos replicate the developmental toxicity phenotype which has been reported in rats.

The chemical composition analysis shows that 3,7,11,15-tetramethyl-2-hexadecen-1-ol occupied 56% and methyl ester of hexadecanoic acid 23% of hexane fraction of leaves of *D. viscosa*. 3,7,11,15-Tetramethyl-2-hexadecen-1-ol is phytol, an acyclic diterpene alcohol. Phytol induced toxicity, cytotoxicity, and genotoxicity in *Allium cepa* at concentrations of 2, 4, 8, and 16 mM [42]. Phytol was reported to regulate the transcription factors PPAR-alpha and retinoid *X* receptor (RXR) [43]. Phytol was identified as a major compound present in the leaf hexane fraction of *Clinacanthus nutans* and it exerted severe toxicity in zebrafish embryos at microgram concentration [44]. Phytol has been graded as category 1 toxic compound which can induce acute and chronic toxicity in aquatic organisms (https://www.caymanchem.com/msdss/17401m.pdf), and the toxicity of D. viscosa which was observed in zebrafish embryos in this study could be due to the presence of phytol as a major compound in the hexane fraction.

**Figure 7:**
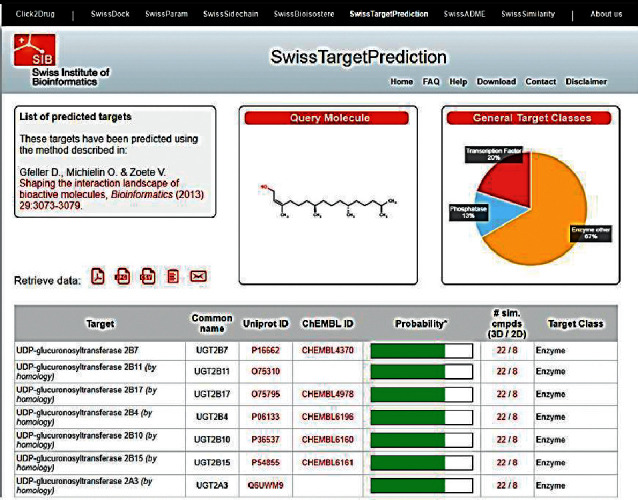
Swiss target protein prediction report of phytol.

The chemical structure of the phytol was searched by Swiss target protein prediction tool (http://old.swisstargetprediction.ch) to identify the possible protein target. As shown in Figure 7, the family of UDP-glucuronosyltransferase was identified as a protein target for phytol.

Family of UDP-glucuronosyltransferases (UGTs) catalyzes the glucuronidation reaction which is a major detoxification pathway in vertebrates. The impairment of UGT results in the accumulation of potentially toxic compounds [45]. Phytol could be one of the natural inhibitors of UGT. The phytol could have impaired the function of UGT in treated embryos which might have resulted in the accumulation of toxic compounds and affected the normal development.

## 6. Conclusion

*Dodonaea viscosa* is a very useful medicinal plant which is being used to treat various diseases in humans. The LD 50 values indicated that the solvent extracts of DV were very toxic in zebrafish embryos and killed developing embryos with concentrations in the microgram range. The morphological abnormalities which were recorded in zebrafish embryo were severe developmental delay, no pigmentation, and severe degenerated notochord which resulted in curved trunk. Moreover, severe cardiac edema and hypertrophy also resulted in treated embryos. The toxicity could be due to impaired UDP-glucuronosyltransferases enzyme due to the presence of major compound phytol (3,7,11,15-tetramethyl-2-hexadecen-1-ol). More detailed studies are needed to check the toxicity of *D. viscosa* in pregnant experimental animals. As *D. viscosa* has been reported to cause the infertility and abortion in female rats, its use and doses must be carefully monitored in pregnant patients.

## Figures and Tables

**Table 1:** GC-MS analysis of *D. viscosa* hexane fraction (DVHF).

Compound name	Chemical formula	Molecular weight (g/mol)	Rt (min)	Area %
(-)-Caryophyllene oxide	C_15_H_24_O	220.35	16.16	1.280
3,7,11,15-Tetramethyl-2-hexadecene	C_20_H_40_	280.5	18.92	2.590
Methyl ester of hexadecanoic acid	C_17_H_34_O2	270.5	19.89	26.330
11,14,17-Eicosatrienoic acid	C_20_H_34_O_2_	306.5	21.62	5.170
3,7,11,15-Tetramethyl-2-hexadecen-1-ol	C_20_H_40_O	296.5	21.74	52.740
Alpha eudesmol	C_15_H_26_O	222	17.13	4.520
Methyl ester of 9,12 octadecanoic acid	C_19_H_34_O_2_	294	21.55	1.630
1,8-Anhydro-Cis alpha-Copaene-8-ol	C_15_H_22_	202	22.30	2.800
(1S,2E,4R,6R,7E,11E,13R)-2,7,11-Cembatriene-4,6,13 triol	C_20_H_34_O_3_	322	26.12	2.940

**Table 2:** Response of zebrafish embryos in terms of mortality by exposure to crude leave extract and different fractions of *D. viscosa*.

Extract	LD50 *µ*g/mL
DV-hex	0.589 ± 0.30
DV crude	0.684 ± 0.74
DV butanol	3.139 ± 0.57
DV chloroform	3.953 ± 0.43

## Data Availability

All the available data are included within the manuscript.
